# Potential Effects of Essential Oils Extracted from Mediterranean Aromatic Plants on Target Weeds and Soil Microorganisms

**DOI:** 10.3390/plants9101289

**Published:** 2020-09-29

**Authors:** Amira Jouini, Mercedes Verdeguer, Samuele Pinton, Fabrizio Araniti, Eristanna Palazzolo, Luigi Badalucco, Vito Armando Laudicina

**Affiliations:** 1 Department of Agricultural, Food and Forestry Sciences, University of Palermo, Viale delle Scienze, Building 4, 90128 Palermo, Italy; amira.jouini@unipa.it (A.J.); eristanna.palazzolo@unipa.it (E.P.); luigi.badalucco@unipa.it (L.B.); 2 Mediterranean Agroforestry Institute (IAM), Universitat Politècnica de València, Camino de Vera s/n, 46022 Valencia, Spain; merversa@doctor.upv.es (M.V.); p.samuele94@gmail.com (S.P.); 3 Department AGRARIA, University Mediterranea of Reggio Calabria, Loc. Feo di Vito, 89100 Reggio Calabria, Italy; fabrizio.araniti@unirc.it

**Keywords:** weed control, phytotoxicity, natural herbicides, microbial biomass, microbial respiration, bacteria, fungi

## Abstract

Essential oils (EOs), extracted from aromatic plants, have been proposed as candidates to develop natural herbicides. This study aimed to evaluate the herbicidal potential of *Thymbra capitata* (L.) Cav., *Mentha × piperita* L. and *Santolina chamaecyparissus* L. essential oils (EOs) on *Avena fatua* L., *Echinochloa crus-galli* (L.) P. Beauv, *Portulaca oleracea* L. and *Amaranthus retroflexus* L. and their effects on soil microorganisms. A pot experiment was set up and three EOs at three doses were applied by irrigation. Efficacy and effects of EOs on weed growth were determined. Soil microbial biomass carbon and nitrogen, microbial respiration, and the main microbial groups were determined at days 7, 28 and 56. EOs demonstrated herbicidal activity, increasing their toxicity with the dose. *T. capitata* was the most effective against all weeds at the maximum dose. *P. oleracea* was the most resistant weed. Soil microorganisms, after a transient upheaval period induced by the addition of EOs, recovered their initial function and biomass. *T. capitata* EO at the highest dose did not allow soil microorganisms to recover their initial functionality. EOs exhibited great potential as natural herbicides but the optimum dose of application must be identified to control weeds and not negatively affect soil microorganisms.

## 1. Introduction

One of the main challenges of the Agriculture of the 21st century is to increase crop production in a sustainable way, e.g., minimizing the use of pesticides [1]. The widespread use of synthetic chemicals may lead to the accumulation of toxic residues in agricultural products and result in soil and groundwater pollution, development of weed resistance, and adverse effects on human and animal health [2,3]. Furthermore, synthetic chemicals can be immobilized in soil by adsorption or binding to colloids [4], affecting both soil organic matter turnover and microbial community composition [5,6,7]. One potential fulfilment to the demand of alternative natural and safe products is the exploitation of renewable resources, such as medicinal and aromatic plants known for their allelopathic properties [8,9].

Plant secondary metabolites, such as essential oils (EOs), include allelochemicals compounds which have been proved to inhibit seed germination and seedling growth [9]. EOs are suitable for sustainable and organic agriculture because of their rapid volatilization and degradation in the environment [10]. Their effectiveness in controlling weeds lies in the joint action of an array of different held compounds, whose quantity and persistence in the environment may be low to inhibit seed germination and plant growth [11,12]. However, when the concentration of one active compound within a given EO is very high, this active compound alone could be effective [13,14,15]. In addition, since EOs usually have various modes of action, it is more complicated for weeds to easily develop resistance against them [16,17]. In fact, EOs can suppress the weed growth by affecting biochemical and physiological processes such as reducing cell survival, chlorophyll and RNA contents; acid soluble carbohydrates; and water-soluble carbohydrates [18,19]. Despite their allelopathic potential, many EOs are classified as “Generally Recognized as Safe” (GRAS) by the US Food and Drug Administration [20].

EOs extracted from Lamiaceae have demonstrated to be effective in inhibiting seed germination in vitro assays [21]. The most important species in this family, in terms of high economical value due to the great production of EOs, are *Mentha × piperita* L. (Peppermint) and *Thymbra capitata* L. (Cav) (synonym *Thymus capitatus* (L.) Hoffmanns. & Link) (Thyme) [22,23]. *Mentha × piperita* L. is a cultivated natural hybrid of *Mentha aquatica* L. (water mint) and *Mentha spicata* L. (spearmint), both native species of the Mediterranean region. *M. Piperita* is cultivated worldwide because its EO has antioxidant and antimicrobial activities and is used as eco-friendly pesticide [24,25]. Likewise, the phytotoxic activity of *M. piperita* EO has been demonstrated in several studies [26,27].

The species of *Thymus* genus, native of Southern Europe, North Africa and Asia [28,29], are largely used as medicinal plants [30]. Due to the presence of polyphenols, *T. capitata* EO is used in food preservation [31] and it has been demonstrated that it possesses antioxidant properties [32]. Moreover, the antimicrobial [33,34] and herbicidal activities [15,35,36] of *T. capitata* EO have been verified. *Santolina chamaecyparissus* L. (cotton lavender) is an aromatic plant belonging to Asteraceae family. Its analgesic, bactericidal, fungicidal, vermifuge, and vulnerary properties have been described [37]. Furthermore, its herbicidal activity is well documented [38]. Although, as aforementioned, some studies have shown that EOs extracted from *M. piperita*, *T. capitata* and *S. chamaecyparissus* may inhibit seed germination and weed growth; the majority of them have been conducted in in vitro conditions and against few weed species. Therefore, their selectivity towards some of the most widespread and troublesome weeds has yet to be investigated. On the other hand, few studies deal with the effects of such EOs on soil microorganisms. Vokou and Liotiri [39] found that EOs extracted from five aromatic plants, not including those tested in this study, increased microbial respiration. Similarly, also EOs extracted from *Lavandula stoechas* L. increased microbial respiration as a result of bacteria growth stimulation [40]. Such results, however, contrast with those of Khare et al. [41] who reported a decrease of microbial biomass and activity. Such few studies with even conflicting results demonstrated that, if EOs were deemed to be used in the field for an integrated pest management, further studies would need to better elucidate their effects on soil microorganisms as playing pivotal roles in the soil organic matter turnover and nutrient cycling. In addition, not all EOs exert the same effect on weeds at a given concentration [35,42].

Therefore, this study had two main purposes: (a) to assess the effectiveness of *M. piperita*, *T. capitata* and *S. chamaecyparissus* EOs to control some of the most troublesome weeds of many crops worldwide including *Avena fatua* L. (wild oat), *Echinochloa crus-galli* (L.) P. Beauv. (barnyard grass), *Portulaca oleracea* L. (common purslane), and *Amaranthus retroflexus* L. (redroot pigweed), and (b) to assess the effect of these EOs on soil microbial biomass C and N, respiration and on the relative abundance of main microbial groups. Such soil biochemical properties were selected because an increase, decrease, or a shift of the main microbial groups are easy to interpret in terms of substrates availability and stress/disturbance for soil microorganisms [43,44,45]. The hypotheses tested were that *T. capitata*, *M. piperita* and *S. chamaecyparissus* EOs could (i) inhibit or, at least, reduce weeds growth; (ii) negatively affect soil microbial biomass C and N and microbial activity; (iii) decrease the fungi to bacteria ratio as the former are less abler than the latter in using available C source; and (iv) determine a stress/disturbance conditions for soil microorganisms.

## 2. Results

### 2.1. Essential Oils Composition

A total of 91 chemicals (Table S2) were identified in the three tested EOs: 17 in *T. capitata*, 35 in *M. piperita* and 39 in *S. chamaecyparissus*. *T. capitata* EO was characterized mainly by a high content of oxygenated monoterpenes (74.0%) and monoterpene hydrocarbons (22.5%; Table 1).

Among the oxygenated monoterpenes (Table S2), carvacrol was the most abundant in *T. capitata EO* (72.3%), menthol in *M. piperita* (51.81%) and 1,8 cineole in *S. chamaecyparissus* (17.50%). Also, *M. piperita* (95.3%) and *S. chamaecyparissus* (39.3%) EOs were particularly rich in oxygenated monoterpenes (Table 1). Among them, menthol (51.8%) and menthone (20.5%) were the most abundant chemicals in *M. piperita*, whereas 1,8-cineole (17.5%) was in *S. chamaecyparissus* (Table S2). The composition of these EOs have been reported and discussed in detail in [15].

### 2.2. Effect of EOs on Target Plants

#### 2.2.1. Effects of EOs on *A. retroflexus*

The efficacy of EOs on *A. retroflexus* was the maximum for *T. capitata* and *M. piperita* EO at the two highest doses (Table 2).

The only two treatments set up for *S. chamaecyparissus* EO (results of S1 treatment are not reported in Table 2 because of the lack of a sufficient number of plants) also suggested an increase of the efficacy with increasing EO dose as efficacy increased from 60 for S2 to 90 for S3. However, regardless of EO type, alive plants showed plant biometric variables lower than those of the two controls (Table 2). The damage level (DL) was the maximum (3) for the two highest doses of *T. capitata* and *M. piperita* and close to them (2.8) for S3; however, also S2 showed higher DL than the two controls.

#### 2.2.2. Effects of EOs on *P. oleracea*

Only *T. capitata* and *M. piperita* EOs, at the two highest doses, were effective to control *P. oleracea*, especially the first EO. However, at the highest doses, all tested EOs affected the plant biometric variables (Table 3).

The damage level followed the same pattern of efficacy, being significantly higher than the two controls only with the two highest doses of *T. capitata* and *M. piperita* EOs.

#### 2.2.3. Effects of EOs on *A. fatua*

This weed species was more effectively controlled by *T. capitata* and *M. piperita* EOs, with efficacy increasing with the dose. *S. chamaecyparissus*, although still significant, was less effective but with S2 and S3 doses not differing between them (Table 4). It was noteworthy that Cf stimulated fresh and dry plant weights.

#### 2.2.4. Effects of EOs on *E. crus-galli*

*T. capitata* EO was the most effective to control this weed, reaching full effectiveness at the highest dose. *M. piperita* EO, at M2 and M3, showed performances similar to T2 and T3, respectively (Table 5).

Otherwise, *S. chamaecyparissus* EO at the highest dose did not even reach half the effectiveness of the other two EOs. Also biometric variables of the remaining alive plants were affected by all EOs, being reduced strongly overall by *T. capitata* and *M. piperita* EOs at the highest doses and to a lesser extent by *S. chamaecyparissus* EO, although the doses the of *T. capitata* were the lowest. The damage level followed an inverse pattern compared to the biometric plant variables being on average higher in *M. piperita* followed by *T. capitata* and then by *S. chamaecyparissus* EO.

#### 2.2.5. Overall Efficacy of EOs

Among the investigated species, *A. retroflexus* and *A. fatua* were the most sensitive targeted weed species to EOs, the efficacy for them being greater than 50, whereas *P. oleracea* and *E. crus-galli* were the least sensitive, with an EOs efficacy lower than 40 (Table 6). Fitoil treatment (Cf) did not exert any phytotoxic effect on weeds since no statistical differences were observed when compared to water control (Cw). All EO treatments, to different extents, significantly provoked weeds death, with, however, *S. chamaecyparissus* EO at the lowest dose causing the least damage (Table 6). *T. capitata* and *M. piperita* EOs were the most effective in killing the targeted weeds.

### 2.3. Effects of EOs on Soil Biochemical Properties and on the Main Microbial Groups

Regardless of incubation day, extractable C (Cextr) and microbial biomass nitrogen (MBN) generally did not differ between Cw and Cf, while microbial biomass carbon (MBC) and metabolic quotient (qCO_2_) were higher in Cf than Cw (Table S3). Consequently, in Cw, the MBC/MBN ratio was more than twice in Cf. The two controls did not differ in respiration rate (Table S3). With regard to the main microbial groups, few differences occurred between the two controls: bacteria did not significantly differ, while fungi and Gram negative bacteria were greater in Cf than in Cw but only at day 56. The above results indicated that fitoil alone exerted some effects on the soil biochemical properties. Thus, in order to isolate the effects due to solely the added EOs, we decided to compare the results of the EOs treated soil with the Cf control.

#### 2.3.1. Effects of *T. capitata* EO on Soil Biochemical Properties and Main Microbial Groups

**Figure 1:**
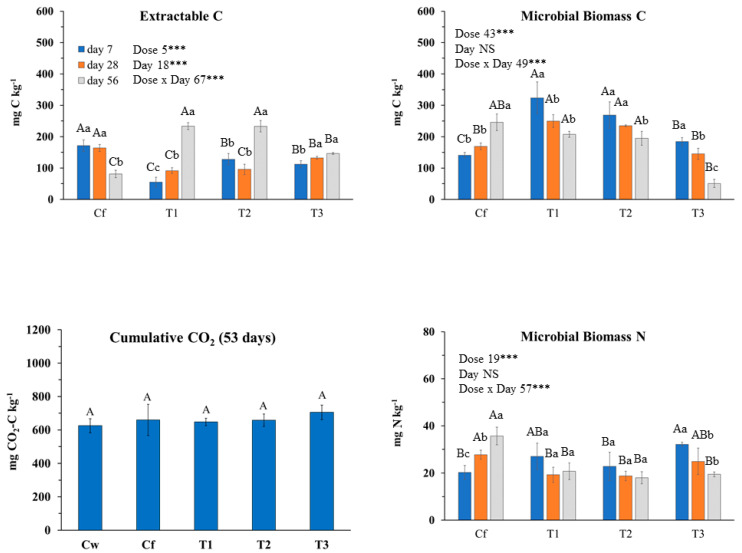
Biochemical soil variables determined at 7, 28 and 56 incubation days after *Thymbra capitata* L. (Cav) essential oil applied by irrigation at different doses: Cw (no Fitoil and EO application), Cf (0.03 μL Fitoil g^−1^ of soil and no EO application), T1 (0.03 μL Fitoil g^−1^ of soil and 0.31 μL EO g^−1^ of soil), T2 (0.03 μL Fitoil g^−1^ of soil and 0.67 μL EO g^−1^ of soil), and T3 (0.03 μL Fitoil g^−1^ of soil and 0.93 μL EO g^−1^ of soil). Reported results are means±standard deviations (*n* = 4). The percentage of variance explained by incubation day, dose of essential oil and by their interaction are also reported. Capital letters indicate significant differences among doses within the same incubation day. Lower case letters indicate significant differences among incubation days within the same dose. *** indicate significant at *p* < 0.001; NS, not significant.

**Figure 2:**
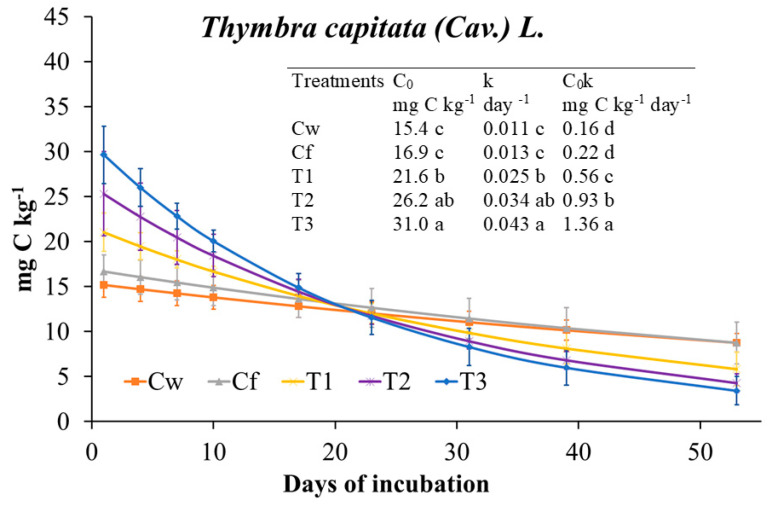
Microbial respiration rate fitted to the exponential first order decay function (Mineralized C = C_0_ e^−kt^) and derived parameters (C_0_, biological available C; k, turnover constant rate; C_0_k, initial potential rate of C mineralization) determined on soil treated with *Thymbra capitata* L. (Cav.) essential oil (EO) applied by irrigation. Treatments were: Cw (no Fitoil and EO application), Cf (0.03 μL Fitoil g^−1^ of soil and no EO application), T1 (0.03 μL Fitoil g^−1^ of soil and 0.31 μL EO g^−1^ of soil), T2 (0.03 μL Fitoil g^−1^ of soil and 0.67 μL EO g^−1^ of soil), and T3 (0.03 μL Fitoil g^−1^ of soil and 0.93 μL EO g^−1^ of soil). Reported results are means (*n* = 4). Bars indicate the standard deviations. Lower case letters indicate significant differences among treatments.

Extractable C was mainly affected by the interaction dose × day (77% of explained variance; Figure 1). During the incubation, it decreased in Cf and increased in soil treated with EO, especially with T1 and T2. MBC trend was opposite compared to Extractable C since throughout incubation it increased in Cf while it decreased in EO-treated soil at any dose. Dose and dose x day affected MBC almost at the same extent (Figure 1). MBC, regardless of incubation day, decreased with increasing EO dose (Figure 1). The interaction dose × day explained the greatest amount of variance of MBN as, during the incubation, it increased in Cf, did not change with T1 and T2 doses, while it decreased in THY3 (Figure 1). Thus, except for T1 and T2 doses, the trend of MBN was parallel to that of MBC. Cumulative CO_2_ was not affected by *T. capitata* EO at any dose (Figure 1); in fact, although at the beginning of the incubation, CO_2_ emission rate was higher in EO treated soils compared to Cf (and Cw), starting from day 23 the rate was inverted (Figure 2). The biologically available C at time zero (C_0_), as well as the turnover constant rate (k), were higher in soil treated with EO than in Cf and proportionally increased with EO dose (Figure 2). The qCO_2_ was significantly affected by both factors (Table 7); indeed, regardless of dose, it was the highest at day 7, whereas at any incubation day with the highest dose. Also, the main microbial groups were generally and similarly affected by both factors and their interaction. In comparison with Cf, *T. capitata* EO slightly decreased bacteria especially at 28 days of the incubation, while fungi strongly decreased with THY3 (Table 7). As a consequence, the F/B ratio in THY1 and THY2 at 56 days was much higher than Cf. BacG+ decreased compared to Cf while BacG− did not differ so much, so that the ratio BacG+/BacG− at 28 days decreased compared to Cf.

#### 2.3.2. Effects of *M. piperita* EO on Soil Biochemical Properties and Main Microbial Groups

**Figure 3:**
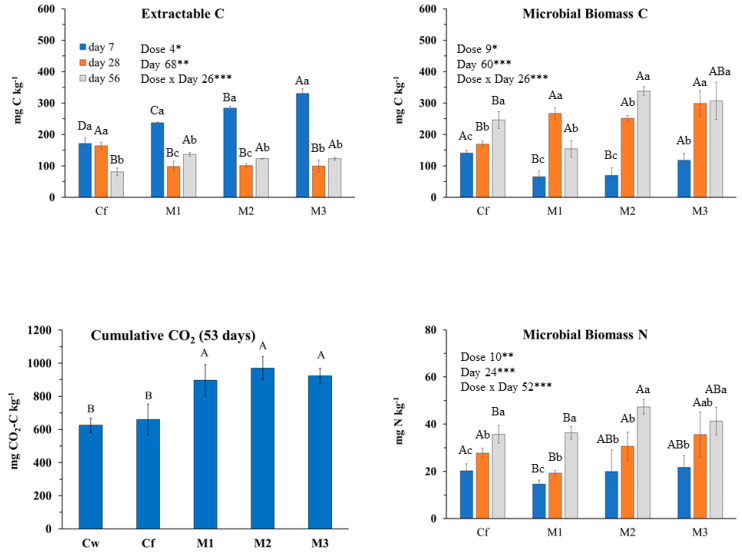
Biochemical soil variables determined at 7, 28 and 56 incubation days after *Mentha × piperita* L. essential oil applied by irrigation at different concentrations: Cw (no Fitoil and EO application), Cf (0.03 μL Fitoil g^−1^ of soil and no EO application), T1 (0.03 μL Fitoil g^−1^ of soil and 0.31 μL EO g^−1^ of soil), T2 (0.03 μL Fitoil g^−1^ of soil and 0.67 μL EO g^−1^ of soil), and T3 (0.03 μL Fitoil g^−1^ of soil and 0.93 μL EO g^−1^ of soil). Reported results are means ± standard deviations (*n* = 4). The percentage of variance explained by incubation day, dose of essential oil and by their interaction are also reported. Capital letters indicate significant differences among doses within the same incubation day. Lower case letters indicate significant differences among incubation days within the same dose. *, ** and *** indicate significant at *p* < 0.05, *p* < 0.01 and *p* < 0.001; NS, not significant.

**Figure 4:**
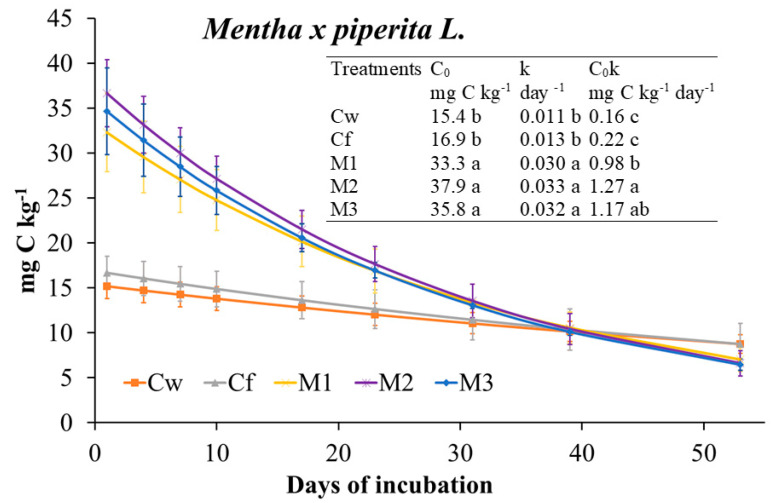
Microbial respiration rate fitted to the exponential first order decay function (Mineralized C = C_0_ e^−kt^) and derived parameters (C_0_, biological available C; k, turnover constant rate; C_0_K, initial potential rate of C mineralization) determined on soil treated with *Mentha × piperita* L. essential oil (EO) applied by irrigation. Treatments were: Cw (no Fitoil and EO application), Cf (0.03 μL Fitoil g^−1^ of soil and no EO application), T1 (0.03 μL Fitoil g^−1^ of soil and 0.31 μL EO g^−1^ of soil), T2 (0.03 μL Fitoil g^−1^ of soil and 0.67 μL EO g^−1^ of soil), and T3 (0.03 μL Fitoil g^−1^ of soil and 0.93 μL EO g^−1^ of soil). Reported results are means (*n* = 4). Bars indicate the standard deviations. Lower case letters indicate significant differences among treatments.

Incubation day more than EO dose affected almost all of the investigated soil biochemical parameters (Figure 3); however, it was noteworthy that at the end of incubation (56 days), changes among Cf and EO treatments were negligible. At day 7, extractable C increased with the EO dose, then strongly decreased, whereas MBC increased with incubation day at the two highest doses (Figure 3). MBN pattern was affected mainly by the interaction of the two tested factors. Indeed, during the first 28 days of the incubation, it was affected only by the lowest dose as it decreased compared to the control. At day 56, it was positively affected only by the medium dose. Compared to Cf, CO_2_ emission rates and, consequently, cumulative CO_2_ were higher in EO treated soils although not significantly different (Figure 3 and Figure 4). Also, the C_0_ and k parameters were the highest in EO-treated soils with no difference among dose. The qCO_2_ was mainly affected by incubation day (69% of variance explained) as it decreased during the incubation at any EO dose (Table 7). Notably, the qCO_2_ at day 7 in EO treated soil was two to three times higher than in Cf, then drastically decreased with negligible differences among EO treatments and control. Both bacteria and fungi slightly decreased compared to the control at any EO dose (Table 7). BacG+ decreased more consistently than BacG− after EO application, so that, at the beginning of the incubation, the ratio BacG+/BacG− was lower than the control. At day 56, however, all the microbial groups did not significantly differ from the control.

#### 2.3.3. Effects of *S. chamaecyparissus* EO on Soil Biochemical Properties and Main Microbial Groups

**Figure 5:**
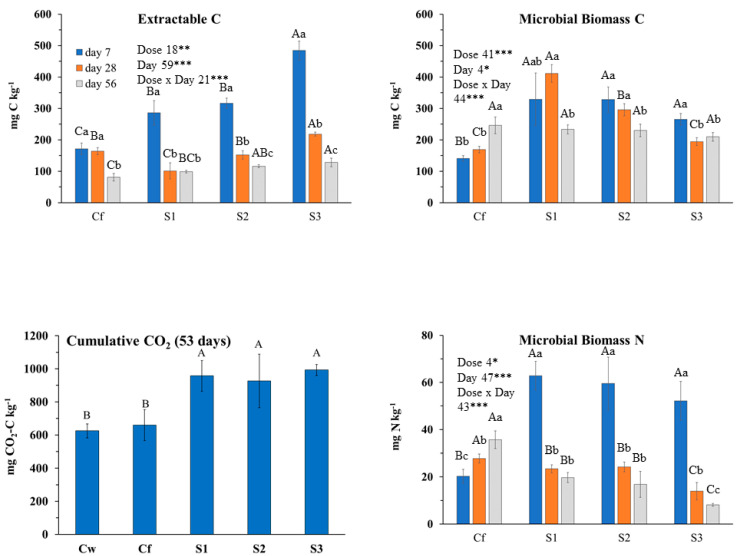
Biochemical soil variables determined at 7, 28 and 56 incubation days after *Santolina chamaecyparissus* L. essential oil applied by irrigation at different concentrations: Cw (no Fitoil and EO application), Cf (0.03 μL Fitoil g^−1^ of soil and no EO application), T1 (0.03 μL Fitoil g^−1^ of soil and 0.31 μL EO g^−1^ of soil), T2 (0.03 μL Fitoil g^−1^ of soil and 0.67 μL EO g^−1^ of soil), and T3 (0.03 μL Fitoil g^−1^ of soil and 0.93 μL EO g^−1^ of soil). Reported results are means±standard deviations (*n* = 4). The percentage of variance explained by incubation day, dose of essential oil and by their interaction are also reported. Capital letters indicate significant differences among doses within the same incubation day. Lower case letters indicate significant differences among incubation days within the same dose. *, ** and *** indicate significant at *p* < 0.05, *p* < 0.01 and *p* < 0.001; NS, not significant.

**Figure 6:**
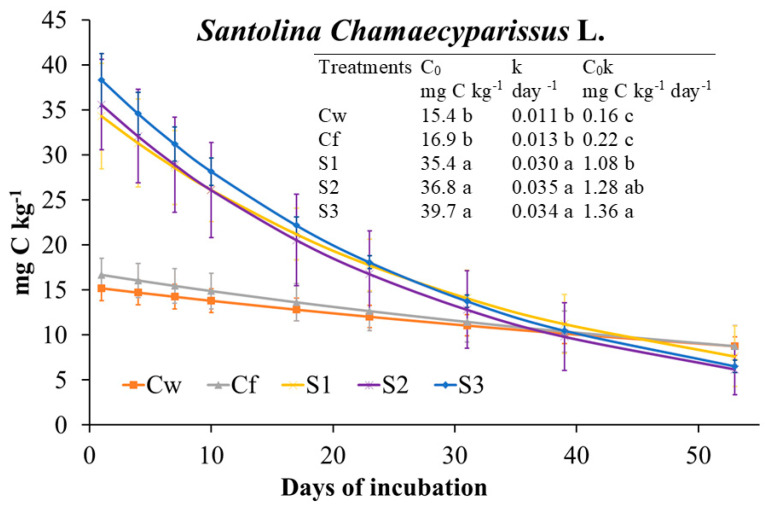
Microbial respiration rate fitted to the exponential first order decay function (Mineralized C = C_0_ e^−kt^) and derived parameters (C_0_, biological available C; k, turnover constant rate; C_0_K, initial potential rate of C mineralization) determined on soil treated with *Santolina chamaecyparissus* L. essential oil (EO) applied by irrigation. Treatments were: Cw (no Fitoil and EO application), Cf (0.03 μL Fitoil g^−1^ of soil and no EO application), T1 (0.03 μL Fitoil g^−1^ of soil and 0.31 μL EO g^−1^ of soil), T2 (0.03 μL Fitoil g^−1^ of soil and 0.67 μL EO g^−1^ of soil), and T3 (0.03 μL Fitoil g^−1^ of soil and 0.93 μL EO g^−1^ of soil). Reported results are means (*n* = 4). Bars indicate the standard deviations. Lower case letters indicate significant differences among treatments.

As in *M. piperita* treatment, incubation day more than EO dose affected investigated soil parameters in *S. chamaecyparissus* treatment (Figure 5). Extractable C decreased in all treatments during the incubation (Figure 5), while at day 7 it increased with the EO dose. Then, it slumped with negligible differences among EO doses and Cf. MBC at day 7 and 28 was higher in EO-treated soil than Cf, also decreasing with the increasing dose, whereas at day 56 any difference among Cf and treated soil disappeared. The trend of MBN was peculiar, since at day 7, in EO-treated soil and regardless of dose, it was about three times higher than in Cf; at day 28, it slumped in the presence of EO down to Cf value with no difference among doses, whereas at day 56, compared to Cf, it decreased with increasing EO dose (Figure 5). Cumulative CO_2_ and other parameters related to C mineralization were higher in EO-treated soil than Cf, with no significant difference among EO doses (Figure 5 and Figure 6). The qCO_2_ was mainly affected by incubation day (Table 7) since at any EO dose it decreased during the incubation, with generally no difference among treatments and the control. Fungi were strongly affected by the EO dose, since during the whole incubation they were increased by SNT1 in comparison to Cf, whereas at days 28 and 56, they were lowered by SNT3 (Table 7). Total bacteria were barely affected by EO, whereas at day 7, BacG+ decreased and BacG− increased; consequently, the BacG+/BacG− ratio strongly increased during the incubation in EO-treated soil (Table 7).

## 3. Discussion

### 3.1. Herbicidal Activity of T. capitata, M. piperita and S. chamaecyparissus EOs

To our knowledge, this is the first study to test in vivo the herbicidal potential of *T. capitata*, *M. piperita* and *S. chamaecyparissus* EOs against the highly competitive and herbicide-resistant weeds *A. retroflexus*, *P. oleracea*, *A. fatua,* and *E. crus-galli* [46]. The herbicidal activity of the tested EOs was dependent on the targeted weed species, the type of EOs (because of their different compositions) and the dose of application. The investigated target weed species showed an overall resistance to the tested EOs according to the following order: *P. oleracea* > *E. crus-galli* > *A. fatua* > *A. retroflexus*. The high ability of *P. oleracea* and *E. crus-galli* in facing chemical-induced stress, adopting molecular, biochemical, and anatomical strategies, is largely reported [47,48,49,50]. Such resistance is dependent on several ecological (e.g., genotype, ecotype) [51,52] and non-ecological factors, such as the chemical used. For example, Norsworthy and Smith [49] reported that *P. oleracea* was significantly resistant to the pre-emergence herbicide dimethenamid but extremely sensitive to pendimethalin. As well as synthetic chemicals, also natural compounds, such as terpenoids, could have a wide range of metabolic targets depending on their molecular structure [53,54]. For example, it has been reported that both the monoterpene citral and the sesquiterpene farnesene are able to alter the hormones balance and cell ultrastructure in seedlings of *Arabidopsis thaliana* [13,55,56], whereas the sesquiterpene trans-caryophyllene alters plant water status, photosystem II efficiency and may also inhibit the germination and growth of several weeds [13]. Overall, these findings suggest that EOs, being a complex mixture of different molecules with different modes of action, could represent an interesting source for the development of new multi-targeted bioherbicides due to their wide and versatile chemical composition. Therefore, it is crucial to know the main chemicals which characterize them.

In *T. capitata* EOs, three chemotypes have been described: thymol, carvacrol, and thymol/carvacrol, each characterized by the predominance of the aforementioned chemicals [57,58,59]. The high abundance of carvacrol and the low amount of thymol, observed in the EO used in our experiments, suggest that it was a carvacrol chemotype. Similarly, the EO of *S. chamaecyparissus*, known to have several chemotypes particularly rich in camphor, borneol, 1,8-cineole and others [60], could be identified as an 1,8 cineole chemotype since this molecule was the most abundant. Finally, as largely reported [61] and confirmed by our analysis, the most abundant chemicals characterizing the EO of *M. piperita* were menthol and menthone. With regard to the applied dose, the herbicidal activity of EOs increased by increasing the dose. *T. capitata* EO was the most effective, but also *M. piperita* EO displayed good potential as a natural herbicide, except against *P. oleracea*. On the other hand, *S. chamaecyparissus* EO revealed a higher selectivity, being very effective especially against *A. retroflexus*. Therefore, although the latter EO was not as potent as *T. capitata* or *M. piperita*, it could be interesting for the development of selective natural herbicides. Regarding the latter issue, it is important to highlight that tested EOs could be an important and sustainable tool for weed management, not only by killing target weeds but also reducing their ability to compete with the crops, as they were capable to reduce their vigor and growth. Many in vitro studies have shown that *T. capitata* EO was able to inhibit seed germination of several noxious weeds [15,35,36,62,63], such as *Erigeron bonariensis*, one of the most important cosmopolite weeds especially in no-tilled soils with problems of resistance to glyphosate [15,35,46]. In addition, *T. capitata* has been described as an allelopathic species capable of reducing both germination and growth of neighboring species [64]. Such allelopathic activity has been ascribed to carvacrol, the main EO constituent [64,65]. Phytotoxicity of pure carvacrol stood out for its extremely high herbicidal activity on *Amaranthus retroflexus* and *Chenopodium album*, which was greater than the commercial herbicide 2,4-D isooctyl ester [66]. Moreover, de Assis Alves et al. [67] demonstrated that carvacrol evidenced a genotoxic effect higher than glyphosate, altering the cell cycle in *Lactuca sativa* and *Sorghum bicolor* meristematic cells.

With regard to the herbicidal activity of *M. piperita* EO, previous studies confirmed the herbicidal potential of its EO against field bindweed (*Convolvulus arvensis* L.), purslane (*Portulaca oleracea* L.) and jungle rice (*Echinochloa colonum* L.), also suggesting its possible use in the formulation of natural herbicides [26]. However, as *M. piperita* EO was phytotoxic for the crops on which it was tested, it could be developed as a non-selective broad spectrum herbicide. Campiglia et al., [68] conducting a pot experiment, found that *M. piperita* EO was effective in controlling the germination of *A. retroflexus*, *Sinapis arvensis*, and *Lolium* spp. These results are consistent with our findings, confirming that *A. retroflexus* is a sensitive species to *M. piperita* EO. The herbicidal activity of *M. piperita* EO is to be ascribed to its ability to interfere with root plasma membrane. Indeed, Maffei et al. [69] have demonstrated that menthol and menthone, the main chemicals held in *M. piperita* EO, are responsible for the depolarization of the membrane potential of cucumber roots. In addition, Mucciarelli et al. [70] demonstrated that both menthol and menthone were significantly reducing mitochondrial respiration in root cells.

About the herbicidal potential of *S. chamaecyparissus* EO, few studies are available, many of which have been performed in vitro. Such studies support the selectivity of this EO depending on the species it is applied against. *S. chamaecyparissus* EO, rich in 1,8-cineole (24.8%), was tested on seed germination and root and shoot growth of four crops (*Zea mays*, *Triticum durum*, *Pisum sativum*, and *Lactuca sativa*) and two weeds (*Portulaca oleracea* and *Vicia sativa*).

The germination of wheat and lettuce was inhibited while it was less harmful for sweet corn and dwarf pea. In addition, it was more active on *P. oleracea*, reducing its shoot and root length, than on the crops [38]. On the other hand, *S. chamaecyparissus* EO from an industrial sample, with 1,8-cineole (9.8%) and 8-methylene-3-oxatricyclo [5.2.0.02,4] nonane (8.2%) as main chemicals, showed a moderate phytotoxicity against the leaf growth of *L. perenne*, but did not show negative effects against *L. sativa* seeds [71].

### 3.2. Changes in Soil Biochemical Properties and in Main Microbial Groups Following the Addition of EOs

Due to the scarcity of studies dealing with the effects of tested EOs on soil biochemical properties and on the main microbial groups, the discussion of the results was carried out in comparison with occurring in vitro studies, when available, aimed at assessing the effects of a single or few key chemicals held within the EOs. Such fact, at the same time, put in evidence the need of further studies to better elucidate the effect of essential oils on soil microorganisms if they are thought to be used as bioherbicides.

#### 3.2.1. *T. capitata* (THY) EO Effects

The addition of THY EO stimulated soil microorganisms but in a different way, depending on the dose and incubation day. The increase of MBC, MBN and respiration, and the decrease of extractable C occurred immediately after the addition of the EO at the lowest dose, suggesting that the available C substrates already in soil, plus those added by EO treatment, were both immobilised and mineralised by soil microorganisms [72]. On the contrary, by increasing the EO dose, at the beginning of incubation, the stimulation concerned only microbial respiration. Towards the end of the incubation, the stimulation effects induced by the two lowest THY EO doses vanished, whereas the highest dose decreased both MBC and activity. These results suggested that THY EO at the highest dose killed part of soil microorganisms (MBC and MBN decreased) and that the surviving ones were not able to use the cytoplasmic materials released outside as demonstrated by the increase of extractable C and the low rate of respiration compared to the control. Overall, such results suggested that THY EO at the highest concentration may be deleterious for soil microorganisms and that such negative effects occur after about 2 months. Such findings agreed with those of Vokou and Liotiri [39] who reported THY EO can be used as a carbon source by soil microorganisms within 1 month since its supply. On the other hand, however, the reduction of microbial biomass agreed with other studies [73,74] who showed that the presence of THY plants strongly interfered with soil microbial biomass and activity.

The biocidal effect of THY EO may be attributed to its main component, carvacrol, which is able to permeate and depolarize the cytoplasmic membrane of microorganisms, so releasing outside the cell membrane associated materials [75,76]. At the beginning of incubation, the changes in MBC and respiration, on average, decreased the qCO_2_ at the two lowest doses while it increased at the highest one towards the end of incubation. Such changes may be attributed to the increase of microbial biomass and the decrease of C substrates availability and the BacG+/BacG− ratio at the two lowest doses, whereas to the increase of C substrates availability and to the decrease of microbial biomass at the highest dose [77,78,79].

#### 3.2.2. *M. piperita* (MNT) EO Effects

MNT EO had an immediate antimicrobial activity as evidenced by the decrease of the soil microbial biomass and by the increase of extractable C at day 7. However, the fraction of soil microorganisms that survived to such initial antimicrobial activity later was able to use the fresh organic C substrates coming form killed microorganisms, as confirmed by the remarkable increase of both microbial biomass and respiration rate but also by the biological available C. Such changes were confirmed by the initial huge increase of the qCO_2_, indicating a strong stress/disturbance condition for soil microorganisms, which was followed by the return to qCO_2_ similar to control at the end of incubation, once the stress finished [79].

The antimicrobial activity of MNT EO could probably be associated with its main constituent, menthol (51.81% in this study). Indeed, İşcan et al., [80] using the bioautographic test, found that menthol was responsible for the antimicrobial activity against plant pathogenic microorganisms.

However, also additive, synergistic or antagonistic effects due to interactions among EO constituents, even those present at low concentrations, cannot be excluded [12,81]. The later increases of MBC and MBN at day 28 and 56 were probably due to both an adaptation to the chemicals added by the EO and to the previous great amount of released organic C substrates, which were immobilized by the surviving microbial biomass, so reducing the stress/disturbance conditions as evidenced by the decrease of qCO_2_. Therefore, such results indicated that MNT EO provoked a transient negative impact on soil microorganisms and/or that soil microorganisms had high resilience capability [82]. Also the effects on the main microbial groups were transient as, after a reduction occurred during the first 28 days of incubation, which concerned both bacteria and fungi, at the end of the incubation, the fungi/bacteria and the BacG+/BacG− ratios did not show significant differences compared to the control.

#### 3.2.3. *S. chamaecyparissus* (SNT) EO Effects

SNT EO stimulated both soil microbial biomass and respiration, but up to 28 days of incubation. However, the simultaneous great increase of extractable C might indicate that immediately after its addition some microorganisms were killed and the released cytoplasmic materials promptly either immobilised or mineralized, as confirmed by the increase of respiration rate and biological available C. The antimicrobial activity of SNT EO was likely due to one of its main constituents, the 1,8-cineole, which is well known for its antimicrobial activity [83,84]. Furthermore, as already stated, the inhibitory activity might result from the interaction of its constituents, even those present at low concentrations. In fact, as demonstrated by Viljoen et al. [85], 1,8-cineole in combination with camphor (4.03% in the SNT EO tested in this study) showed higher antimicrobial effects. The great stimulation induced by SNT EO, however, did not affect the qCO_2_, since MBC and respiration rate were proportionally enhanced. Such stimulation effect was transient since at the end of the incubation both MBC and respiration were not significantly different from the control. On the other hand, MBN was significantly decreased at the end of the incubation, suggesting, at least, an increase in the fungi-to-bacteria ratio since the first have higher MBC/MBN ratio than the latter [86,87]. PLFAs data partially confirmed such findings, the fungi/bacteria ratio being higher than the control at the lowest EO concentrations and day 7. Moreover, PLFAs data put in evidence that the relative abundances of the main microbial groups at the end of the incubation rarely changed, also with no relapses on the microbial activity, so suggesting a good resilience of the soil microorganisms.

## 4. Materials and Methods

### 4.1. Essential Oils

*Thymbra capitata* (L.) Cav., *Mentha* × *piperita* L. and *Santolina chamaecyparissus* L. EOs were purchased from Bordas (Sevilla, Spain), Sigma-Aldrich (Darmstadt, Germany) and Ecoaromuz (Ademuz, Valencia, Spain), respectively. The analysis of the EOs was carried out by gas chromatography with flame ionization detector (GC-FID) and mass spectrometry (GC-MS). A Clarus 500 GC (Perkin-Elmer Inc., Wellesley, PA, USA) chromatograph equipped with a FID detector and capillary column ZB-5 (30 m × 0.25 mm i.d. × 0.25 μm film thickness; Phenomenex Inc., Torrance, CA, USA) was used for quantitative analysis. The injection volume was 1 μL. The GC oven temperature was set at 60 °C for 5 min, with increases of 3 °C per min up to 180 °C, and then increases of 20 °C per min up to 280 °C, which was maintained for 10 min. Helium was the carrier gas (1.2 mL min^−1^). Injector and detector temperatures were set at 250 °C. The percentage composition of the EO was calculated from GC peak areas without correction factors by means of the software Total Chrom 6.2 (Perkin-Elmer Inc., Wellesley, PA, USA). Analysis by GC-MS was performed using a Clarus 500 GC-MS (Perkin-Elmer Inc., Wellesley, PA, USA) apparatus equipped with the same capillary column, carrier and operating conditions described above for GC-FID analysis. Ionization source temperature was set at 200 °C, and 70 eV electron impact mode was employed. MS spectra were obtained by means of total ion scan (TIC) mode (mass range m/z 45–500 uma). The total ion chromatograms and mass spectra were processed with the Turbomass 5.4 software (Perkin-Elmer Inc., Wellesley, PA, USA). Retention indexes (RIs) were determined by injecting C_8_–C_32_ n-alkanes standards under the same conditions. The EO components were identified by comparison of calculated RIs and high probability matches according to mass spectra computer library search (NIST MS 2.0) and available data from literature. Identification of α-pinene, β-pinene, camphene, myrcene, limonene, camphor, terpinolene, β-thujone, borneol, terpinen-4-ol, bornyl acetate, and linalool was confirmed by comparison of their experimental RI with those of the reference standards (Sigma-Aldrich, Milan, Italy).

### 4.2. Herbicidal Activity of EOs against Target Weeds

Seeds of *Portulaca oleracea* L., *Amaranthus retroflexus* L. and *Avena fatua* L. were purchased from Herbiseed (Reading, United Kingdom) in 2017, and seeds of *Echinochloa crus-galli* (L.) P. Beauv. were collected from rice fields in Sollana (Valencia, Spain) in September 2017. Seeds germination was achieved using a germination-growth chamber (Equitec, Spain). *P. oleracea*, *A. retroflexus* and *E. crus-galli* seeds were germinated using a 16 h/8 h (light/dark) photoperiod, settling the temperatures at 30 ± 0.1 °C and 20 ± 0.1 °C for light and dark conditions, respectively. *A. fatua* seeds were germinated using 8 h (23.0 ± 0.1 °C)/16 h (18.0 ± 0.1 °C) light/dark conditions. Germination conditions were established based in previous works [36]. After germination (about 1 week), emerged seedlings were selected for uniformity in growth and individually transplanted in polypropylene square pots (8 × 8 × 7 cm) previously filled with a 2 cm drainage layer of perlite and a 5 cm layer of soil (220 g) collected in an organic citrus orchard (39°37′24.8″ N, 0°17′25.6″ W, Puzol, Valencia, Spain). For each treatment, 10 repetitions (10 pots) were prepared, being in total 430 pots for all treatments, including the controls. The pots of the same treatment were located in the same tray and then transferred to a glass greenhouse separately to avoid possible mixing of the treatments. Dates during which weeds were grown, and greenhouse temperature and humidity conditions, are reported in Table S1.

To each pot, 80 mL of water were added to bring the soil to 4/5 of its water holding capacity (WHC), and left overnight. The day after, 100% of soil WHC was reached by irrigation from the top by adding 20 mL of an emulsion containing a given EO [88]. EO water emulsions were prepared using 0.5 mL L^−1^ of the emulsifier Fitoil (Xeda, Italy). On the basis of the results of previous studies [15,35,46], three different emulsions were prepared for each EO at the following concentrations:

- *T. capitata*: 4 (T1), 8 (T2), 12 (T3) µL mL^−1^;
- *M. piperita*: 12 (M1), 16 (M2), 20 (M3) µL mL^−1^;
- *S. chamaecyparissus*: 12 (S1), 16 (S2), 20 (S3) µL mL^−1^.

Also, two controls were established: The first irrigated only with water (Cw) and the second irrigated with water plus Fitoil at 0.5 mL L^−1^ (Cf). EOs treatments were applied once when plants reached the phenological stage of two to three true leaves, corresponding to 12–13 BBCH (Biologische Bundesanstalt, Bundessortenamt and CHemical industry) scale for the monocotyledons *A. fatua* and *E. crus-galli*, and three to four true leaves, corresponding to 13–14 BBCH scale for the dicotyledons *P. oleracea* and *A. retroflexus*. In order to evaluate any phytotoxic effect, photos of the plants were taken just after 24 and 48 h after the application of the treatments and then each 3 or 5 days for the whole experiment. At the end of the experiment, the entire plant from each pot was reclaimed by dipping in water the root apparatus to remove any soil residues and images of all plants were registered. The software Digimizer v.4.6.1 (MedCalc Software, Ostend, Belgium, 2005–2016) was used to process and analyze the images to determine total (TL), root (RL) and aerial part (APL) length of the plants and also the damage level (DL). The weeds were analyzed in different stages, depending on the weed species. For *A. retroflexus*, it was when the control plants had five to seven true leaves and the inflorescence was visible (51 stage BBCH scale), for *P. oleracea* when the control plants had more than seven pairs of leaves and the first side shoot was visible (21 stage BBCH scale), for *A. fatua* when the control plants had seven leaves developed (17 stage BBCH scale), and for *E. crus-galli* when the control plants had five leaves developed (15 stage BBCH). We had to refer to control plants because treated plants were abnormal and damaged. The damage level was assessed developing a damage scale for each species. The scale range was from 0 (no damage) to 4 (death of the plant) for the monocotyledons (*A. fatua* and *E. crus-galli*; Figures S1 and S2, respectively), and from 0 (no damage) to 3 (death of the plant) for the dicotyledons (*P. oleracea* and *A. retroflexus*; Figures S3 and S4, respectively). Fresh (FW) and dry weights (at 60 °C for 48 h; DW) were also determined. The efficacy of a given EO was considered as its capacity to kill the plants and was assessed by attributing the value 0 if the plant was alive and 100 if the plant was dead.

### 4.3. Effects of EOs on Soil Microorganisms

To test the effects of EOs on soil microorganisms, a short-term laboratory incubation experiment was set-up. The topsoil (0–15 cm) of a citrus [*Citrus sinensis* (L.) Osbeck] orchard (38°06′31.34″ N, 13°21′03.45″ W, Palermo, Italy) never treated with synthetic chemicals was used. Its main characteristics were: sand 64.9%, clay 15.9%, organic carbon 2.3%, pH 7.0, electric conductivity 0.1 dS m^−1^, and total nitrogen 1.2 g kg^−1^. After sampling, the soil was air-dried and sieved at 2 mm. Aliquots of 350 g of soil were placed in 1L plastic bottles and moistened with only water up to 2/3 of 50% of its WHC. Then, a volume of EO emulsion was added thus reaching the 50% of its WHC. The amounts of EO added were 31 (THY1), 62 (THY2) and 93 μL 100 g^−1^ (THY3) of soil for *T. capitata* treatment, and 93, 123 and 153 μL 100 g^−1^ of soil for both *M. piperita* (MNT1, MNT2, MNT3) and *S. chamaecyparissus* (SNT1, SNT2, SNT3) treatments. Two controls were also prepared: The first with only water (Cw) and the second with water and fitoil (Cf) at a concentration of 0.05% (*v*/*v*) to moisten the soil. Four replicates per treatment were run. After the EOs addition, plastic bottles were incubated in the dark, at constant temperature (25.0 ± 0.5 °C), for 56 days. During the incubation, water loss was monitored by weighing the bottles and eventually watering them with only water to maintain the soil WHC at 50%.

At days 7, 28 and 56, soils were analyzed to determine some biochemical properties. The fumigation–extraction method [89] was used to assess microbial biomass C (MBC). Fumigated and not fumigated soil sub-samples (15 g) were extracted with 0.5 M K_2_SO_4_, at a ratio of 1:4 (*w*/*v*). Total organic C in soil extracts was determined by hot digestion-oxidation (sulphuric acid-dichromate mixture). MBC was estimated as the difference between the organic C held in fumigated extract and that in non-fumigated extract, multiplied by a conversion factor (kEC) of 2.64. The K_2_SO_4_-extractable C of not fumigated soil was assumed as a proxy of the readily available C pool [90]. Microbial biomass N (MBN) was calculated multiplying by 5 the difference between the ninhydrin reactive N determined on fumigated and non-fumigated soil 0.5 M K_2_SO_4_ extracts, respectively, according to Joergensen and Brookes [91]. Concurrently, glass jars of 200 mL with 20 g of soil aliquots from each of the above treatment were incubated, in the dark and at 23–25 °C, to determine microbial respiration. The CO_2_ accumulated in the headspace of the glass jars at days 1, 4, 7, 10, 17, 23, 31, 39, and 53 was assessed by a gas chromatograph equipped with a thermal conductivity detector. At each CO_2_ determination, jars were ventilated with fresh air for 30 min and then sealed again, after possible replenishment of lost soil moisture by distilled water. The C mineralization rate, expressed as mg CO_2_–C kg^−1^ dry soil day^−1^, was fitted to the following first order decay function:

Mineralized C = C_0_ e^−kt^

where C_0_ is the biologically available C (mg kg^−1^) at time zero (i.e., the intercept value), k is the decay rate constant, and t is the sampling incubation day.

The total CO_2_–C mineralized over 59 days of incubation was calculated according to Ioppolo et al. [92] by the linear interpolation of two neighboring rates and the integration over time:

$$\mathrm{Total}\;C\;\mathrm{mineralized}=\;\sum_{i=1}^{n-1}\;\left(r_{i}+\;r_{i+1}\right)*\frac{d_{i}}{2}$$

where *i* and *i* + 1 are the first and the last of two close CO_2_–C rate measurements; *n* is the last day of measurement of CO_2_–C rate; *r* is the CO_2_–C rate expressed as mg CO_2_–C kg^−1^ dry soil day^−1^; and *d* is the number of days between the two consecutive CO_2_ rate measurements.

The specific respiration rate, or metabolic quotient (qCO_2_), i.e., the amount of CO_2_ emitted per unit of MBC per time unit, was calculated as mg CO_2_–C g^−1^ MBC h^−1^.

Fatty acids (FAs) were extracted from soils according to the modified Bligh and Dyer method [93]. The fatty acid methyl esters (FAMEs) were detected by a gas chromatograph (FOCUS™ GC, Thermo Scientific Inc., Waltham, MA USA) equipped with a flame ionization detector and a fused-silica capillary column Mega-10 (50 m × 0.32 mm I.D.; film thickness 0.25 μm). The GC temperature progression was initial isotherm at 115 °C for 5 min, increases of 1.5 °C per min up to 230 °C, and final isotherm at 230 °C for 2 min. Both injection port and detector were set at 250 °C, and helium at 1 mL min^−1^ in a constant flow mode was used as a carrier. The injected volume was 1 μL (50:1 split ratio). Nonadecanoic acid methyl ester (19:0; cat no. N-5377, Sigma-Aldrich Co., Milan Italy) was used as internal standard for the quantification of FAMEs. Peak identification was done by comparing the retention times of each FAMEs to known standards (Supelco Bacterial Acid Methyl Esters mix cat no. 47080-U and Supelco 37 Component FAME mix cat no. 47885-U). Fatty acids with less than 14 carbon atoms or more than 20 carbon atoms were excluded as considered originating from non-microbial sources. The FAs i15:0, a15:0, 15:0, i16:0, i17:0, 17:0, cy17:0,18:1ω7, and cy19:0 were used to represent bacterial biomass while using 18:2ω6,9 for fungal biomass. The fungal-to-bacterial ratio (F/B), i.e., a measure of what proportion of the microbial community is bacteria compared to the proportion of the microbial community that is fungi, was calculated. The FAs i15:0, a15:0, i16:0, and i17:0 were chosen to represent Gram-positive bacteria (BacG+) while 16:1ω7, 18:1ω7, cy17:0 and cy19:0 for Gram-negative bacteria (BacG−) [44,45]. Fatty acids were designated as the total number of carbon atoms followed by a colon, the number of double bonds followed by the position of the double bond from the aliphatic (ω) end of the molecule. The prefixes a and i indicate ante-iso and iso branching, respectively, and “cy” a cyclopropane group.

### 4.4. Statistical Analysis

The plant experiment was carried out in a completely randomized design because our unit of experimentation was the pot (10 repetitions for each treatment), and the treatments were assigned to the pots in a random way. After the application of the treatments, the pots with the same treatment were placed in a tray in order to avoid the loss of the treatments by lixiviation when the pots were irrigated. The pots were considered always as singular units and all the variables were measured individually for each pot. Biometric plant variables (TL, RL, APL, FW, DW) and DL data were evaluated for normality and variance homogeneity and then subjected to one-way ANOVA, followed by Fisher’s multiple comparison test (LSD intervals, Least Significant Difference, at *p* < 0.05) for the separation of the means in each species. A multifactor analysis of variance (ANOVA) was performed on efficacy including species and treatment as effects.

Reported soil data, referred to as oven-dry soil (105 °C) weight, are the arithmetic means of four replicates. Before performing parametric statistical analyses, normal distribution and variance homogeneity of the data were checked by Kolmogorov–Smirnoff goodness-of-fit and Levene’s tests, respectively. Within each EO treatment, soil data were subjected to two-way ANOVA with EO dose (four levels; three EO doses and the control, Cf) and incubation day (three levels: days 7, 28 and 56) as factors. Within each EO type (THY, MNT, SNT), significant differences at *p* < 0.05 among doses at the same incubation day and among incubation days at the same dose were assessed by the least significant difference (LSD) post-hoc test. For both biometric plant and soil variables, ANOVA was carried out without any transformation of the data. All analyses were performed by Statgraphics Centurion version XVII.

## 5. Conclusions

Several studies have been carried out on the phytotoxic activity of EOs against weeds and on their potential use as natural herbicides. The majority of these works have been performed in vitro experiments and not in microcosms that try to mimic the natural conditions. Moreover, in vitro approaches seeds and/or seedlings are directly exposed to the EOs in sterile conditions, i.e., strongly reducing and/or retarding EOs transformation/degradation normally mediated by soil microorganisms. To our knowledge, this is the first time that the effects of essential oils from *T. capitata*, *M. piperita and S. chamaecyparissus* against targeted weeds and soil microorganisms have been studied with a more practical approach, i.e., in vivo conditions, monitoring their effects in order to know their real potential as an alternative to synthetic chemicals, within a strategy of Integrated Weed Management and analyzing the benefits or disadvantages derived from their employment.

Results clearly demonstrated that tested EOs, to a different extent, were significantly effective against weeds, killing them completely or reducing significantly their growth parameters. Among them, *T. capitata* was the most effective, followed by *M. piperita*. Both EOs showed a broad spectrum of activity, with *T. capitata* at the highest doses applied (12 μL mL^−1^) killing plants of all weed species (100 efficacy), except for *P. oleracea* (90 efficacy). *M. piperita* at the highest dose (20 μL mL^−1^) controlled completely (100 efficacy) *A. retroflexus* and *A. fatua* plants but showed 90 and 40 efficacy on *P. oleracea* and *E. crus-galli*, respectively. Although *S. chamaecyparissus* EO was less active compared with the other EOs, it displayed a very remarkable selective activity, being highly effective against *A. retroflexus* (90 efficacy at the highest dose, 20 μL mL^−1^). It could be interesting to study it more profoundly as a selective herbicide, while *T. capitata* and *M. piperita* could have a wider action, exhibiting excellent potential for the development of broad-spectrum herbicides. A good natural herbicide, besides being effective, at the same time should not have side effects on soil microorganisms. Here, results clearly demonstrated that, except for *T. capitata* EO at the highest concentration, which significantly increased the specific respiration rate, the other EOs generally stimulated soil biochemical properties, or their effect on them was transient. Furthermore, even when changes in the main microbial groups persisted, soil microbial activity was not irredeemably affected, suggesting that essential oils did not compromise the functional redundancy.

Since EOs are able to decrease the weed growth parameters by reducing their fitness and competitiveness, another advantage in using these EOs, from a conservationist point of view in agro-ecosystems, could be that to maintain a high biodiversity by not completely eradicating the weeds, instead giving the crop an opportunity to outcompete them. 

## Figures and Tables

**Table 1:** Abundance (%) and number (in parenthesis) of grouped chemical compounds identified in the essential oils extracted by hydrodistillation from *T. capitata*, *M. piperita* and *S. chamaecyparissus*.

Group of Chemical Compounds	*T. capitata*	*M. piperita*	*S. chamaecyparissus*
Monoterpene hydrocarbons	22.54 (9)	1.95 (10)	9.30 (8)
Oxygenated monoterpenes	73.98 (6)	95.35 (16)	39.32 (14)
Sesquiterpene hydrocarbons	3.14 (1)	2.22 (5)	21.78 (8)
Oxygenated sesquiterpenes	0.14 (1)	0.00 (0)	15.64 (6)
Others	0.00 (0)	0.14 (4)	12.91(3)

**Table 2:** Effects of *T. capitata* (T1, T2 and T3 are 4, 8 and 12 µL mL^−1^), *M. piperita* (M1, M2 and M3 are 12, 16 and 20 µL mL^−1^), and *S. chamaecyparissus* (S1, S2 and S3 are 12, 16 and 20 µL mL^−1^) essential oils applied by irrigation against *A. retroflexus* on the efficacy (E), plant biometric variables [aerial part (APL), root (RL) and total length (TL), fresh (FW) and dry weights (DW)] and damage level (DL). Cw (only water, neither Fitoil nor EO application), Cf (0.03 μL Fitoil g^−1^ of soil, no EO application). The damage level (DL) was assessed developing a damage scale ranging from 0 (no damage) to 3 (death of the plant). The efficacy (E) of a given EO was considered as its capacity to kill the plants and was assessed by attributing the value 0 if the plant was alive and 100 if the plant was dead. Both DL and E are dimensionless. Data are the arithmetic mean of 10 replicates.

Treatment/Dose	E	APL cm	RL cm	TL cm	FW g	DW g	DL
Cw	0 d	9.9 a	7.4 a	17.3 a	0.48 a	0.09 a	0.2 c
Cf	0 d	11.4 a	6.5 a	17.9 a	0.46 a	0.08 a	0.1 c
T1	30 c	5.9 b	2.6 b	8.5 b	0.32 ab	0.04 b	1.3 b
T2	100 a	0 e	0 c	0 d	0 d	0 c	3.0 a
T3	100 a	0 e	0 c	0 d	0 d	0 c	3.0 a
M1	50 bc	5 bc	2.9 b	8.1 b	0.27 bc	0.04 b	1.6 b
M2	100 a	0 e	0 c	0 d	0 d	0 c	3.0 a
M3	100 a	0 e	0 c	0 d	0 d	0 c	3.0 a
S1	-	-	-	-	-	-	-
S2	60 b	2.9 cd	1.7 bc	4.5 bc	0.13 cd	0.02 bc	2.0 b
S3	90 a	0.8 de	0.4 c	1.2 cd	0.03 d	0 c	2.8 a

Different letters along the column indicate significant differences among treatments at *p* < 0.05.

**Table 3:** Effects of *T. capitata* (T1, T2 and T3 are 4, 8 and 12 µL mL^−1^ dose of essential oil), *M. piperita* (M1, M2 and M3 are 12, 16 and 20 µL mL^−1^ dose of application), and *S. chamaecyparissus* (S1, S2 and S3 are 12, 16 and 20 µL mL^−1^ dose of application) essential oils applied by irrigation against *P. oleracea* on the efficacy (E), plant biometric variables [aerial part (APL), root (RL) and total length (TL), fresh (FW) and dry weights (DW)] and damage level (DL). Cw (only water, neither Fitoil nor EO application), Cf (0.03 μL Fitoil g^−1^ of soil, no EO application). The damage level (DL) was assessed developing a damage scale ranging from 0 (no damage) to 3 (death of the plant). The efficacy (E) of a given EO was considered as its capacity to kill the plants and was assessed by attributing the value 0 if the plant was alive and 100 if the plant was dead. Both DL and E are dimensionless. Data are the arithmetic mean of 10 replicates.

Treatment/Dose	E	APL cm	RL cm	TL cm	FW g	DW g	DL
Cw	0 c	9.3 a	10.9 a	19.1 a	1.6 a	0.20 a	0 c
Cf	0 c	8.9 a	11.3 a	20.2 a	1.5 a	0.17 a	0 c
T1	0 c	8.3 ab	8.5 abc	16.9 ab	1.3 ab	0.14 ab	0 c
T2	40 b	4.9 cd	5.5 cd	10.4 de	0.7 c	0.07 c	1.2 b
T3	90 a	0.7 e	0.7 e	1.3 f	0.1 d	0 e	2.7 a
M1	30 bc	6.0 bc	6.1 bcd	12.1 bcd	1.0 bc	0.10 bc	0.9 bc
M2	40 b	5.1 cd	5.3 cd	10.7 cde	0.7 c	0.08 c	1.2 b
M3	40 b	3.0 de	2.8 de	5.7 ef	0.6 cd	0.05 ce	1.2 b
S1	0 c	8.0 ab	8.4 abc	16.4 abc	1.6 ab	0.16 a	0 c
S2	10 bc	7.9 ab	8.7 abc	16.7 abc	1.6 ab	0.16 a	0.3 bc
S3	30 bc	5.8 bc	6.1 bcd	11.9 bcd	0.9 bc	0.08 bc	0.9 bc

Different letters along the column indicate significant differences among treatments at *p* < 0.05.

**Table 4:** Effects of *T. capitata* (T1, T2 and T3 are 4, 8 and 12 µL mL^−1^ dose of essential oil), *M. piperita* (M1, M2 and M3 are 12, 16 and 20 µL mL^−1^ dose of application), and *S. chamaecyparissus* (S1, S2 and S3 are 12, 16 and 20 µL mL^−1^ dose of application) essential oils applied by irrigation against *A. fatua* on the efficacy (E), plant biometric variables [aerial part (APL), root (RL) and total length (TL), fresh (FW) and dry weights (DW)] and damage level (DL). Cw (only water, neither Fitoil nor EO application), Cf (0.03 μL Fitoil g^−1^ of soil, no EO application). The damage level (DL) was assessed developing a damage scale ranging from 0 (no damage) to 4 (death of the plant). The efficacy (E) of a given EO was considered as its capacity to kill the plants and was assessed by attributing the value 0 if the plant was alive and 100 if the plant was dead. Both DL and E are dimensionless. Data are the arithmetic mean of 10 replicates.

Treatment/Dose	E	APL cm	RL cm	TL cm	FW g	DW g	DL
Cw	0 e	28.0 a	18.1 a	46.1 a	1.02 b	0.19 b	0.15 d
Cf	0 e	28.5 a	17.7 a	46.2 a	1.45 a	0.23 a	0.15 d
T1	80 abc	6.0 cd	3.3 c	10.4 cd	0.20 d	0.02 d	3.2 ab
T2	90 ab	2.4 cd	1.8 c	4.2 cd	0.09 d	0.02 d	3.6 ab
T3	100 a	0 d	0 c	0 d	0 d	0 d	4.0 a
M1	70 bc	5.0 cd	4.1 c	9.2 cd	0.09 d	0 d	3.0 b
M2	90 ab	1.4 d	0.5 c	1.9 cd	0.01 d	0 d	3.7 ab
M3	100 a	0 d	0 c	0 d	0 d	0 d	4.0 a
S1	30 d	16.3 b	9.3 b	25.6 b	0.57 c	0.09 b	1.4 c
S2	60 c	8.8 c	3.7 c	12.6 c	0.16 d	0.02 d	2.9 b
S3	60 c	5.0 cd	3.5 c	8.5 cd	0.06 d	0.01 d	3.1 ab

Different letters along the column indicate significant differences among treatments at *p* < 0.05.

**Table 5:** Effects of *T. capitata* (T1, T2 and T3 are 4, 8 and 12 µL mL^−1^ dose of essential oil), *M. piperita* (M1, M2 and M3 are 12, 16 and 20 µL mL^−1^ dose of application), and *S. chamaecyparissus* (S1, S2 and S3 are 12, 16 and 20 µL mL^−1^ dose of application) essential oils applied by irrigation against *E. crus-galli* on the efficacy (E), plant biometric variables [aerial part (APL), root (RL) and total length (TL), fresh (FW) and dry weights (DW)] and damage level (DL). Cw (only water, neither Fitoil nor EO application), Cf (0.03 μL Fitoil g^−1^ of soil, no EO application). The damage level (DL) was assessed developing a damage scale ranging from 0 (no damage) to 4 (death of the plant). The efficacy (E) of a given EO was considered as its capacity to kill the plants and was assessed by attributing the value 0 if the plant was alive and 100 if the plant was dead. Both DL and E are dimensionless. Data are the arithmetic mean of 10 replicates.

Treatment/Dose	E	APL cm	RL cm	TL cm	FW g	DW g	DL
Cw	0 d	28.6 a	20.8 a	49.5 a	1.20 a	0.15 a	0.10 f
Cf	0 d	27.3 ab	19.9 a	47.2 a	0.90 ab	0.11 ab	0.10 f
T1	10 cd	26.1 ab	17.2 ab	43.3 ab	0.91 ab	0.10 ab	0.9 e
T2	50 b	14.6 cd	9.9 cd	24.5 cd	0.40 cde	0.06 bcde	2.4 bc
T3	100 a	0 f	0 f	0 f	0 f	0 f	4.0 a
M1	40 bc	10.4 cde	7.8 cde	18.5 cde	0.21 def	0.03 def	1.8 cd
M2	50 b	6.1 def	5.6 def	11.7 def	0.16 ef	0.02 ef	3.7 ab
M3	90 a	2.3 ef	2.5 ef	4.8 ef	0.21 f	0 f	4.0 a
S1	10 cd	19.2 bc	13.1 bc	32.3 bc	0.70 bc	0.1 bc	0.8 de
S2	30 bcd	17.2 c	12.3 bc	29.5 bc	0.54 cd	0.1 bcd	1.8 cd
S3	40 bc	12.2 cd	6.9 cde	19.1 cde	0.32 def	0.04 cdef	2.3 bc

Different letters along the column indicate significant differences among treatments at *p* < 0.05.

**Table 6:** Overall essential oils (EOs) efficacy per species and per treatment. Treatments were: *T. capitata* EO (T1, T2 and T3 are 4, 8 and 12 µL mL^−1^ doses), *M. piperita* EO (M1, M2 and M3 are 12, 16 and 20 µL mL^−1^ doses) and *S. chamaecyparissus* EO (S1, S2 and S3 are 12, 16 and 20 µL mL^−1^ doses) applied by irrigation. Cw (only water, neithr Fitoil nor EO application), Cf (0.03 μL Fitoil g^−1^ of soil, no EO application). The efficacy (E) of a given EO was considered as its capacity to kill the plants and was assessed by attributing the value 0 if the plant was alive and 100 if the plant was dead (it is dimensionless). Data are the arithmetic mean of 10 replicates.

Species	Efficacy
*Amaranthus retroflexus*	64 a
*Portulaca oleracea*	25 c
*Avena fatua*	52 a
*Echinochloa crus-galli*	38 b
Treatment/Dose	Efficacy
Cw	0 g
Cf	0 g
T1	30 ef
T2	70 bc
T3	97 a
M1	47 d
M2	70 bc
M3	82 ab
S1	13 fg
S2	40 de
S3	55 cd

Different letters along the column indicate statistical differences among species or treatments at *p* < 0.05.

**Table 7:** Metabolic quotient, main microbial groups (nmol g^−1^) and percentage of variance explained by incubation day, dose of essential oil and by their interaction. Treatments were: *T. capitata* (THY1, THY2 and THY3 are 4, 8 and 12 µL mL^−1^ doses), *M. piperita* (MNT1, MNT2 and MNT3 are 12, 16 and 20 µL mL^−1^ doses) and *S. chamaecyparissus* (SNT1, SNT2 and SNT3 are 12, 16 and 20 µL mL^−1^ doses) essential oils applied by irrigation; Cf, control with fitoil. Data are the arithmetic mean of four replicates.

Treatment/Dose	Day	qCO_2_	Bacteria	Fungi	BacG+	BacG−	F/B	BacG+/BacG−
Cf	7	4.2 a	76 a	13 b	32 a	44 a	0.17 a	0.72 a
Cf	28	2.8 b	85 a	19 a	43 a	41 a	0.22 a	1.06 a
Cf	56	1.5 c	82 a	17 ab	47 a	36 b	0.23 a	1.28 a
THY1	7	2.4 Ca	67 Ba	15 Ab	30 ABa	37 Ba	0.22 Bb	0.81 Aa
	28	1.8 Bb	69 Aa	16 Aab	30 Aa	39 Aa	0.23 Ab	0.77 Aab
	56	1.2 Bc	16 Ab	20 Aa	6 Ab	9 Ab	1.31 Aa	0.71 Bb
THY2	7	3.2 Ba	55 Ba	12 Ba	24 Ba	31 Ba	0.22 Bb	0.91 Ab
	28	1.8 Bb	64 Aa	13 Ba	27 Aa	36 Aa	0.20 Ab	0.74 Ab
	56	0.9 Bc	32 Ab	13 ABa	19 Aa	13 Aa	1.04 Aa	1.55 Aa
THY3	7	5.2 Aa	97 Aa	3 Cb	35 Aa	61 Aa	0.03 Ba	0.57 Aa
	28	2.7 Ab	46 Bb	4 Cb	20 Bb	27 Ba	0.08 Bab	0.74 Aa
	56	2.9 Ab	60 Aab	9 Ba	20 Ab	40 Aa	0.18 Ba	0.69 Ba
Day		59 ***	16 **	12 *	NS	22 **	34 **	14 *
Dose		22 **	19 **	72 ***	27 **	14 *	19 **	22 **
Day × Dose		14 *	29 **	6 *	26 **	24 **	31 **	29 **
MNT1	7	15.0 Aa	72 Aa	7 Bb	15 ABc	58 Aa	0.11 Bb	0.28 Ac
	28	2.3 Bb	73 Aa	10 Bab	32 Ab	41 Ab	0.13 Cab	0.77 Ab
	56	1.9 Ab	58 Aa	10 Ba	39 Aa	20 ABc	0.18 Bb	2.56 ABa
MNT2	7	15.8 Aa	59 Aa	11 Ab	13 Bc	46 Aa	0.20 Aa	0.31 Ac
	28	2.5 Bb	62 Ba	12 ABab	30 ABb	32 Bb	0.20 Ba	0.96 Ab
	56	0.8 Bc	55 Aa	13 Ba	41 Aa	15 Bc	0.24 Ba	2.81 Aa
MNT3	7	10.3 Ba	66 Aa	14 Ab	18 Ac	48 Aa	0.21 Ab	0.39 Ac
	28	2.0 Bb	61 Bb	15 Ab	26 Bb	34 Bab	0.25 Ab	0.76 Ab
	56	0.9 Bc	62 Aab	23 Aa	31 Aa	31 Ab	0.37 Aa	1.02 Ba
Day		69 ***	NS	18 **	51 ***	53 ***	24 **	52 ***
Dose		7 *	44 ***	54 ***	23 **	5 *	45 ***	5 *
Day × Dose		22 **	NS	15 *	NS	12 *	8 *	25 **
SNT1	7	3.9 Ba	81 Aa	27 Aa	4 Bc	78 Aa	0.33 Aa	0.05 Bc
	28	1.6 Cb	93 Aa	23 Ab	44 Ab	49 Ab	0.24 Ab	0.93 Ab
	56	1.4 Ab	74 ABa	21 Ab	68 Aa	6 Bc	0.30 Aab	11.29 Aa
SNT2	7	3.7 Ba	80 Ac	19 Ba	10 Ac	70 Aa	0.23 ABa	0.15 Ab
	28	2.0 Bb	90 Ab	17 Bab	39 ABb	51 Ab	0.19 Bab	0.76 Ab
	56	1.1 Ac	100 Aa	16 Bb	62 Aa	38 Ac	0.16 Bb	1.73 Ca
SNT3	7	4.9 Aa	66 Ac	9 Ca	10 Ac	56 Ba	0.14 Ba	0.18 Ab
	28	3.3 Ab	86 Aa	3 Cb	36 Bb	50 Aa	0.03 Cb	0.72 Ab
	56	1.3 Ac	56 Bb	2 Cb	48 Aa	8 Bb	0.03 Cb	5.85 Ba
Day		81 ***	14 *	NS	72 ***	61 ***	5 *	40 ***
Dose		7 *	25 **	82 ***	NS	6 *	72 ***	13 *
Day × Dose		NS	25 **	10 *	16 **	28 **	13 *	44 ***

Capital letters indicate significant differences among doses at the same incubation day within a treatment. Lower case letters indicate significant differences among incubation days at the same dose within a treatment. Numbers in bold indicate significant differences at *p* < 0.05 compared to the fitoil control (Cf) at a given day. BacG+ and BacG− indicate Gram positive and negative bacteria, respectively. *, ** and *** indicate significance at *p* < 0.05, *p* < 0.01 and *p* < 0.001; NS, not significant. *n* = 4.

## Supplementary Materials

The following are available online at https://www.mdpi.com/2223-7747/9/10/1289/s1, Table S1: Greenhouse temperature and relative humidity conditions during the experimental period, Table S2: Chemical composition of essential oils extracted by hydrodistillation from *T. capitata* (TC), *M. piperita* (MP) and *S. chamaecyparissus* (SC). KI, Kovats index, Table S3: Biochemical parameters and main microbial groups determined in soil irrigated with water (Cw) or with fitoil emulsion (Cf, 0.5 mL L^−1^) during the incubation.
