# Supplementary material for: Potential Effects of Essential Oils Extracted from Mediterranean Aromatic Plants on Target Weeds and Soil Microorganisms
Source: Plants (Basel). 2020 Sep 29;9(10):1289. doi: 10.3390/plants9101289 (PMC7600404; doi:10.3390/plants9101289)
Supplement: Supplementary file 1 [file plants-09-01289-s001.pdf]

plants

MDPI

Table S1 Greenhouse temperature and relative humidity conditions during the experimental period

Species	Date	Temperature (°C)			Relative Humidity (%)		
		Mean	Maximum	Minimum	Mean	Maximum	Minimum
A. retroflexus	30/08/2018-28/09/2018	26.8	38.0	20.0	71.3	89.0	31.14
P. oleracea	3/07/2018-22/07/2018	29.0	44.0	23.2	64.0	94.0	17.4
A. fatua	26/05/2018-24/06/2018	26.0	37.7	19.0	62.3	97.5	23.2
E. crus-galli	16/07/2018-25/07/2018	28.8	44.0	23.2	64.3	92.0	17.4

Table S2 Chemical composition of essential oils extracted by hydrodistillation from *T. capitata* (TC), M. *piperita* (MP) and *S. chamaecyparissus* (SC). KI, Kovats index.

Compounds	KI.	TC	MP	SC
**Monoterpene hydrocarbons (%)**		22.54	1.95	9.30
Santolina triene	908	-	-	0.13
α-Thujene	930	0.89	0.01	-
α-Pinene	938	0.74	0.28	0.85
Thuja-2,4(10)-diene	947	-	-	-
Camphene	951	-	-	0.28
Sabinene	975	-	0.14	0.17
β-Pinene	978	0.29	0.43	3.98
Myrcene	991	1.95	0.01	-
α-Phellandrene	1004	0.16	-	-
γ-Terpinene	1016	**7.77**	0.13	1.18
α-Terpinene	1016	1.61	-	0.69
p-Cymene	1025	**8.93**	0.18	2.01
Limonene	1029	0.20	0.73	-
(Z)-β-Ocimene	1040	-	0.03	-
iso-Terpinolene	1087	-	0.02	-
**Oxygenated monoterpenes (%)**		73.98	95.35	39.32
1,8-Cineole	1031	0.11	4.31	**17.50**
trans-Pinocarveol	1037	-	-	0.17
Artemisia ketone	1062	-	-	4.63
(Z)-Sabinene hydrate	1070	-	0.76	-
Linalool	1097	0.77	0.09	0.42
Camphor	1142	-	-	4.03
Menthone	1154	-	**20.52**	-
(E)-Pinocamphone	1159	-	-	0.18
(Z)-Chrysanthemol	1162	-	-	3.80
Menthofuran	1163	-	5.21	-
neo-Menthol	1165	-	3.12	-
Borneol	1168	0.16	-	1.11
(Z)-Pinocamphone	1172	-	-	2.03
Menthol	1175	-	**51.81**	-
Terpinen-4-ol	1177	0.37	0.67	2.69
iso-menthol	1182	-	0.60	-
Neoisomenthol	1187	-	0.08	-
α-Terpineol	1188	-	0.17	0.21
Myrtenal	1192	-	-	1.31
Myrtenol	1193	-	-	1.07
Verbenone	1198	-	-	0.16
m-Cumenol	1230	-	-	-
Pulegone	1236	-	0.83	-
Piperitone	1251	-	0.32	-
neo-Menthyl acetate	1273	-	0.16	-
p-Menth-1-en-7-al	1279	-	-	-
Menthyl acetate	1291	-	**6.56**	-
Thymol	1292	0.27	-	-
Carvacrol	1300	**72.30**	-	-
iso-Menthyl acetate	1303	-	0.16	-
**Sesquiterpene hydrocarbons (%)**		3.14	2.22	21.78
α-Ylangene	1373	-	-	0.08
α-Bourbonene	1381	-	0.17	-

β-Caryophyllene	1415	3.14	1.47	0.39
β-Farnesene	1454	-	0.02	-
allo-Aromadendrene	1457	-	-	4.23
trans-Cadina-1(6),4-diene	1473			0.36
Germacrene-D	1477	-	0.42	**12.60**
β-Selinene	1491	-	0.13	-
Elixene	1492	-	-	2.80
γ-Cadinene	1509	-	-	0.32
δ-Cadinene	1519	-	-	1.00
**Oxygenated sesquiterpenes (%)**		0.14	0.00	15.64
Bornyl acetate	1283	-	-	0.08
Spathulenol	1477	-	-	1.42
Caryophyllene oxide	1577	0.14	-	0.19
Viridiflorol	1587	-	-	**13.56**
β-Oplopenone	1602	-	-	0.16
α-Cadinol	1649	-	-	0.23
**Others (%)**		0.00	0.14	12.91
1-Butanol. 2-methyl-, propanoate	973	-	-	0.20
1-Octen-3-ol	980	-	0.02	-
3-Octanol	995	-	0.07	-
iso-Amyl 2-methyl butyrate	1101	-	0.02	-
n-Amyl isovalerate	1106	-	0.04	0.48
8-methylene-3-oxatricyclo[5,2,0,0(2,4)]nonane	1117	-	-	**12.24**
**TOTAL IDENTIFIED (%)**		**99.80**	**99.66**	**98.95**

Table S3 Biochemical parameters and main microbial groups determined in soil irrigated with water (Cw) or with fitoil emulsion (Cf, 0.5 mL L^{-1}) during the incubation.

Treatment	Water Control (Cw)			Fitoil Control (Cf)		
Sampling day	7	28	56	7	28	56
Soil variables						
Cextr (mg kg^{-1})	136 Ba	152 Aa	69 Ab	171 Aa	164a A	81 Ab
MBC (mg kg^{-1})	219 Ac	316 Ab	460 Aa	141 Bb	169 Bb	246 Ba
MBN (mg kg^{-1})	14 Bb	21 Aab	28 Aa	20 Ab	28 Aab	36 Aa
MBC/MBN	15.9 Aa	15.5 Aa	16.2 Aa	7.0 Ba	6.1 Ba	6.9 Ba
Microbial respiration (mg CO_2-C kg^{-1} d^{-1})	15.5 Aa	11.9 Aab	8.7 Ab	14.3 Aa	11.4 Aab	8.8 Ab
Metabolic quotient (qCO_2; mg CO_2-C g^{-1} MBC h^{-1})	3.0 Ba	1.6 Bab	0.8 Bb	4.2 Aa	2.8 Aab	1.5 Ab
Bacteria (nmol kg^{-1})	65.2 Aa	73.5 Ba	66.8 Ba	76.5 Aa	84.7 Ba	82.4 Ba
Fungi (nmol kg^{-1})	13.8 Aa	18.6 Aa	2.3 Bb	13.3 Aa	18.8 Aa	17.3 Aa
Bacteria gram positive (nmol kg^{-1})	20.7 Ac	36.4 Ab	53.1 Aa	32.0 Ab	43.5 Aa	46.7 Aa
Bacteria gram negative (nmol kg^{-1})	44.4 Aa	37.1 Aa	13.7 Bb	44.5 Aa	41.2 Aa	35.7 Aa
Fungi/Bacteria	0.21 Aa	0.25 Aa	0.04 Bb	0.17 Aa	0.22 Aa	0.23 Aa
Bacteria Gram positive/ Gram negative	0.48 Ab	1.00 Ab	4.14 Aa	0.72 Aa	1.06 Aa	1.28 Bb

Capital letters indicate significant differences among the two controls at the same incubation day. Lower case letters indicate significant differences among incubation days within a given control. N = 4.

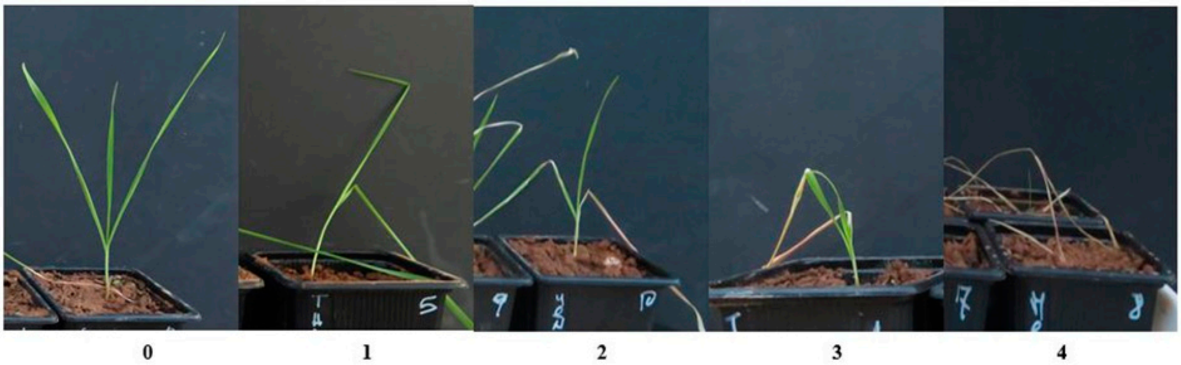

Figure S1. Scale of damage level for the monocotyledon *Avena fatua*.

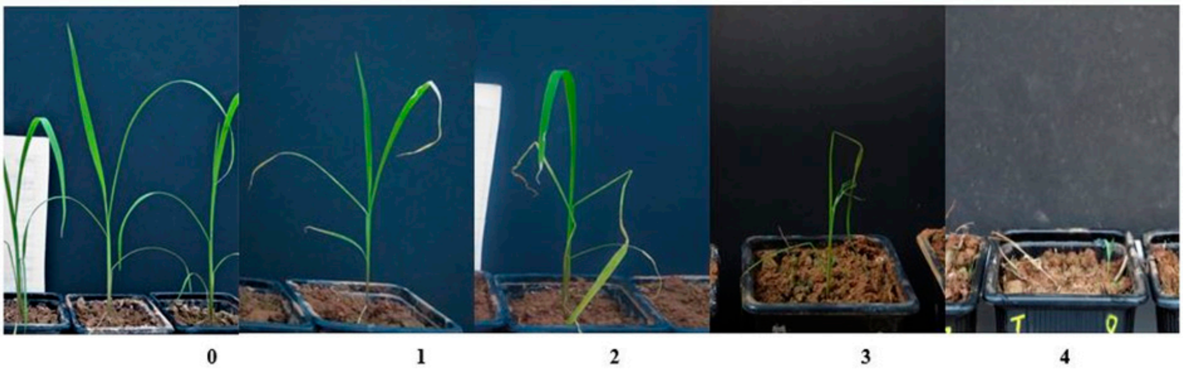

Figure S2. Scale of damage level for the monocotyledon *Echinochloa crus-galli*.

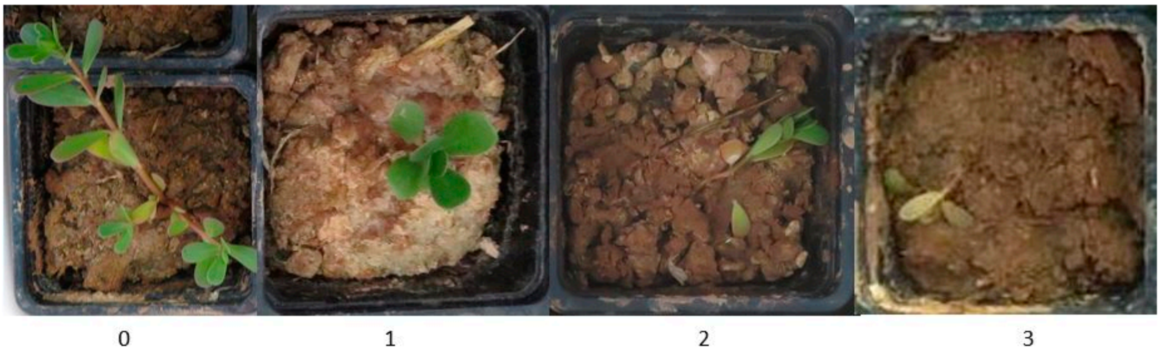

Figure S3. Scale of damage level for the dicotyledon *Portulaca oleracea*.

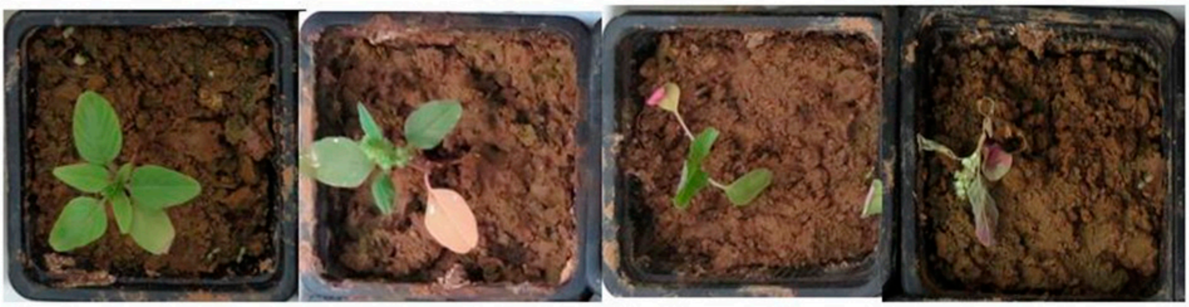

0 1 2 3

Figure S4. Scale of damage level for the dicotyledon *Amaranthus retroflexus.*
